# Beneficial Outcomes of Omega-6 and Omega-3 Polyunsaturated Fatty Acids on Human Health: An Update for 2021

**DOI:** 10.3390/nu13072421

**Published:** 2021-07-15

**Authors:** Ivana Djuricic, Philip C. Calder

**Affiliations:** 1 Department of Bromatology, Faculty of Pharmacy, University of Belgrade, 11221 Belgrade, Serbia; ivana.djuricic@pharmacy.bg.ac.rs; 2 School of Human Development and Health, Faculty of Medicine, University of Southampton, Southampton SO16 6YD, UK; 3 NIHR Southampton Biomedical Research Centre, University Hospital Southampton NHS Foundation Trust and University of Southampton, Southampton SO16 6YD, UK

**Keywords:** omega-6 fatty acids, omega-3 fatty acids, inflammation, oxidative stress, COVID-19

## Abstract

Oxidative stress and inflammation have been recognized as important contributors to the risk of chronic non-communicable diseases. Polyunsaturated fatty acids (PUFAs) may regulate the antioxidant signaling pathway and modulate inflammatory processes. They also influence hepatic lipid metabolism and physiological responses of other organs, including the heart. Longitudinal prospective cohort studies demonstrate that there is an association between moderate intake of the omega-6 PUFA linoleic acid and lower risk of cardiovascular diseases (CVDs), most likely as a result of lower blood cholesterol concentration. Current evidence suggests that increasing intake of arachidonic acid (up to 1500 mg/day) has no adverse effect on platelet aggregation and blood clotting, immune function and markers of inflammation, but may benefit muscle and cognitive performance. Many studies show that higher intakes of omega-3 PUFAs, especially eicosapentaenoic acid (EPA) and docosahexaenoic acid (DHA), are associated with a lower incidence of chronic diseases characterized by elevated inflammation, including CVDs. This is because of the multiple molecular and cellular actions of EPA and DHA. Intervention trials using EPA + DHA indicate benefit on CVD mortality and a significant inverse linear dose–response relationship has been found between EPA + DHA intake and CVD outcomes. In addition to their antioxidant and anti-inflammatory roles, omega-3 fatty acids are considered to regulate platelet homeostasis and lower risk of thrombosis, which together indicate their potential use in COVID-19 therapy.

## 1. Introduction

**Figure 1:**
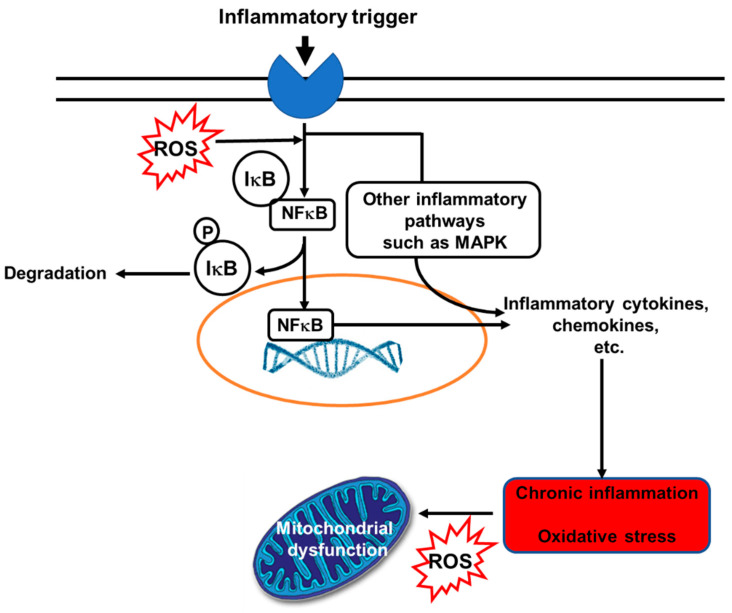
The bidirectional links between inflammation and oxidative stress. Reactive oxygen species (ROS) can act as inflammatory trigger initiating inflammation. On the other hand, inflammation induces oxidative stress. Abbreviations used: IkB, inhibitory subunit of NFkB; MAPK, mitogen-activated protein kinase; NFkB, nuclear factor kappa-light-chain-enhancer of activated B cells; P, phosphate; ROS, reactive oxygen species. Reproduced from [7].

Despite the COVID-19 pandemic with more than 3.4 million deaths in the last year due to infection with severe acute respiratory syndrome coronavirus 2, chronic non-communicable diseases (NCDs) are still easily the most common global cause of morbidity and mortality. The World Health Organization (WHO) estimated that 41 million deaths in 2018 were due to NCDs [1]. NCDs include cardiovascular diseases (CVDs), cancers, non-infectious respiratory diseases and metabolic diseases. Alongside other risk factors, oxidative stress and inflammation have been recognized as important contributors to risk of NCDs. Elevated levels of inflammatory markers, including several cytokines and chemokines, are seen in those with NCDs [2]. Inflammation is an important part of host defense, firstly by creating a hostile environment for microbes and later by initiating tissue repair, recovery, and maintenance of homeostasis. However, prolonged (unresolved) inflammation and continuous release of pro-inflammatory mediators can cause tissue damage, metabolic changes and loss of function [3,4,5]. Thus, inflammation is a “double-edged sword”. Likewise, in low or medium concentrations, free radicals (a term used to describe reactive oxygen species (ROS) and reactive nitrogen species (RNS)) have physiological roles in protecting cells from various harmful influences, including microbes. Furthermore, free radicals have a regulatory function in intracellular signaling cascades in several cell types, such as endothelial cells, fibroblasts, cardiomyocytes, and thyroid tissue [6]. However, when production of free radicals exceeds a particular concentration and disturbs the cell redox potential, adverse effects are manifested. For example, many cellular structures can be damaged as a result of oxidative stress, including membranes, proteins, lipids, lipoproteins and DNA [6]. Inflammation and oxidative stress are inter-related: oxidative stress can activate inflammatory signaling pathways, while inflammation induces oxidative stress (Figure 1 [7]).

**Figure 2:**
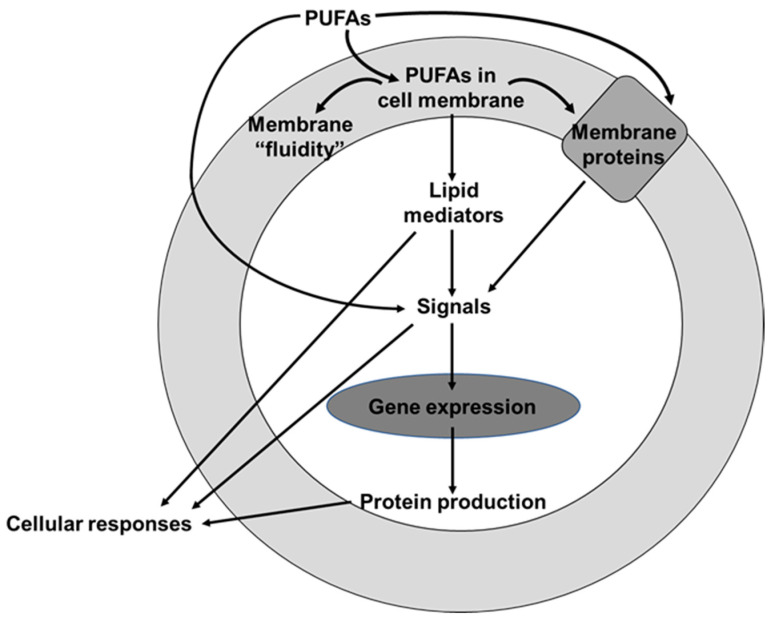
Schematic overview of how polyunsaturated fatty acids (PUFAs) affect cell responses.

Fatty acids are an integral component of cell membrane phospholipids, with specific functional, metabolic, and signaling roles [8]. Different cells have different fatty acid compositions that influence membrane fluidity and flexibility, and the function of membrane proteins [8]. Different intakes of polyunsaturated fatty acids (PUFAs) result in different levels of PUFAs in cell membrane phospholipids from where they exert actions on cell functions and cell and tissue responsiveness to signals (Figure 2). PUFAs may act as antioxidants by regulating the antioxidant signaling pathway [9,10] and may modulate inflammatory processes [10,11]. This review will provide an update on knowledge on PUFAs and their effects on human health.

## 2. Omega-6 and Omega-3 PUFAs

**Figure 3:**
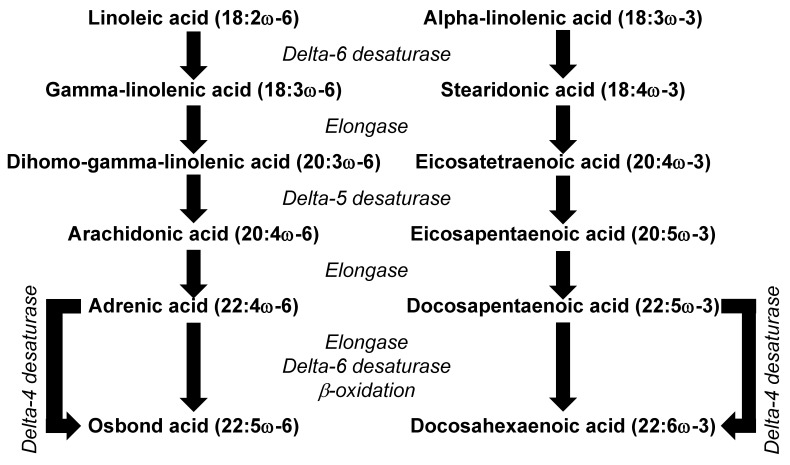
Pathway of conversion of essential fatty acids to their more unsaturated and longer chain derivatives.

There are two main families of PUFAs that are relevant to human health, the omega-6 and the omega-3 PUFAs. In most diets, the PUFAs present in the highest amounts are linoleic acid (LA, 18:2ω-6) and α-linolenic acid (ALA, 18:3ω-3). LA and ALA are not synthesized in animals and so are regarded as essential fatty acids. Because they are synthesized in plants, LA and ALA are mainly found in high proportions in foods of plant origin. For example, many seeds, nuts, and plant oils are rich in LA; these include safflower, sunflower and pumpkin seeds; walnuts; and corn, sunflower, safflower and soybean oils. Pumpkin seeds, walnuts and soybean oil are also good sources of ALA, as are flax seeds and flaxseed oil. The essentiality of PUFAs was first described in studies in rats, where deficiency resulted in a number of serious symptoms [12]. Essential fatty acid deficiency has been rarely described in humans, but low intakes are said to contribute to dermatitis, renal hypertension, mitochondrial activity disorders, CVDs, type 2 diabetes, impaired brain development, arthritis, depression, and decreased body resistance to infection [13]. Some of these defects may be due to low intake of LA and ALA. However, they may also be due to low levels of the metabolic products of LA and ALA, including the long-chain omega-6 PUFA arachidonic acid (AA, 20:4ω-6) and the long-chain omega-3 PUFAs eicosapentaenoic acid (EPA, 20:5ω-3) and docosahexaenoic acid (DHA, 22:6ω-3). The metabolic conversion of LA to AA and of ALA to EPA shares the same enzymes (Figure 3), which means that the rate of synthesis of AA and EPA will depend upon the relative availability of the substrate (i.e., LA and ALA). The typical Western diet is characterized by 5 to 15 times higher intake of LA than ALA [14], meaning that LA is the dominant substrate for the pathway. This might explain why the metabolism of ALA to EPA and DHA appears to be limited in humans [14] and why the levels of AA in blood and in many cell types greatly exceed the levels of EPA and DHA [15].

## 3. Omega-6 PUFAs and Human Health

### 3.1. General Overview

LA may have physiological actions and health benefits in its own right or through acting as the substrate for the synthesis of other omega-6 PUFAs including γ-linolenic acid (GLA, 18:3ω-6), dihomo-γ-linolenic acid (DGLA, 20:3ω-6) and AA (see Figure 3). It is considered that 1–2% of daily energy intake as LA is required for normal growth and development. LA, GLA, DGLA and AA can all be incorporated into cell membrane phospholipids, although AA is typically present in the highest amounts [15]. This is of physiological significance, because AA is a substrate for bioactive lipid mediators, many of which are together referred to as eicosanoids (see later).

### 3.2. Omega-6 PUFAs and the Skin

LA has a specific and unique role in the structural integrity of the skin and in barrier function because it is as an essential constituent of ceramides [16]. The epidermis consists of cells and a lipid-rich extracellular matrix (with 50% ceramides, 25% cholesterol and 15% free fatty acids) [17]. The extracellular matrix forms the stratum corneum permeability barrier, whose fluidity depends on the LA content, and other fatty acids seem not to be able to substitute for LA in this role. Due to a lack of the required enzymes, the skin has limited conversion of LA to AA [18]. Fatty acids can be delivered to the epidermis by cellular uptake through lipoprotein receptors, and subsequently they may act to protect the function and appearance of the skin and to modulate the inflammatory response [19,20]. Different PUFAs may relieve symptoms associated with inflammatory skin disorders (e.g., atopic dermatitis (AD)/eczema; psoriasis), most likely through changes in the ratio of pro-and anti-inflammatory eicosanoids [18,21,22]. AD is long-term inflammation of the skin with complex and multifactorial pathophysiology. The Th2 inflammatory pathway dominates in the acute phase of AD with the elevated release of interleukins (IL)-4, -5, -13, and -31, followed by activation of mast cells and eosinophils and production of specific immunoglobulin E antibodies. A change in the cytokine profile accompanies the progression of acute to chronic skin inflammation, including a move away from the Th2 phenotype towards the Th1, Th22 and Th17 phenotypes [22]. PUFAs may affect skin inflammation by acting as substrates for lipid mediators, such as eicosanoids (see later), which are directly involved in inflammatory processes [23,24], and through modulation of immune cell function and cytokine production via eicosanoids or other mechanisms. Prevention of active inflammation and the improvement of epidermal barrier function may be an excellent therapeutic approach for patients with AD. It seems that treatment with combined omega-6 PUFAs (LA oils and GLA as a dietary supplement) and long-chain omega-3 PUFAs (particularly EPA and DHA) has the potential to ameliorate inflammatory processes in the skin [18], helping with disease management.

### 3.3. Linoleic Acid, Blood Cholesterol and CVDs

It has been established that moderate LA intake, as a partial replacement for saturated fatty acids, reduces blood total cholesterol and low-density lipoprotein (LDL)-cholesterol concentrations [25,26]. This seems to be the result of the upregulation of hepatic LDL receptor (LDLR) gene and protein expression, thereby promoting hepatic clearance of circulating LDL [26]. LA enhances the transcription of the liver X receptor alpha (LXRα) gene, probably via peroxisome proliferator-activated receptors (PPARs). In turn, LXRα upregulates the expression of the cholesterol 7α-hydroxylase (CYP7) gene which encodes the enzyme regulating the pathway of the conversion of cholesterol into bile acids. Therefore, LA helps to catabolize cholesterol by promoting CYP7 activity [26]. In response to cholesterol use in hepatocytes (for bile acid synthesis), the number and activity of LDLRs is increased [26]. This involves sterol regulatory element binding proteins (SREBPs), which are transcription factors with an essential role in regulating cholesterol and triglyceride levels in the body [27]. SREBPs activate the transcription of genes that encode proteins involved in cholesterol uptake (i.e., LDLR) and in cholesterol, fatty acid, and triglyceride synthesis [27]. SREBPs are produced as proteins that require cleavage for activation: this process is facilitated by SREBP cleavage-activating protein (SCAP) which binds native SREBPs, transporting them to the Golgi for proteolytic processing [28,29]. Thus, SCAP acts to promote SREBP cleavage and so regulates SREBP activity. Sterols bind to SCAP and prevent the SCAP–SREBP complex from leaving the endoplasmic reticulum, thus hindering proteolytic cleavage of the native SREBP and acting to control hepatic cholesterol homeostasis [29]. Thus, the SCAP pathway plays a crucial role in the feedback regulation of hepatic lipid metabolism [28,29]. By acting to remove hepatic cholesterol through bile acid synthesis, LA will promote SREBP activation (via SCAP activity), leading to upregulation of LDLR expression, so favoring LDL-cholesterol clearance from the circulation.

One more benefit of LA on circulating LDL-cholesterol is its inhibitory effect on proprotein convertase subtilisin kexin type 9 (PCSK9) [30], which directly interacts with LDLR [31]. Secreted PCSK9 binds to LDLR, promoting its internalization. By lowering PCSK9, LA helps to maintain the LDLR on the hepatocyte membrane, and so favoring LDL-cholesterol clearance. Genetic variants in LDLR, PCSK9 and apolipoprotein B genes correlate with an atherogenic lipid profile and consequently worse cardiovascular outcomes [32]. Among 232 unrelated Japanese patients, 6% had LDLR/PCSK9 gene variants and higher levels of LDL-cholesterol than those with only LDLR gene variants [32]. Further, these patients had a greater risk of nonfatal myocardial infarction [32].

The ability of LA to lower LDL-cholesterol would be expected to result in lowered CVD incidence and mortality. A systematic review and meta-analysis of prospective cohort studies identified that the replacement of 5% energy as saturated fatty acids by LA was associated with a 9% reduction in coronary heart disease (CHD) [33]. In another, more recent meta-analysis of 30 prospective cohort studies, higher circulating and adipose tissue LA (which reflect the intake of LA) were both associated with lower risk of major cardiovascular events [34]. Thus, cohort studies demonstrating lower CVD with higher LA intake agree with the LDL-cholesterol lowering by LA which occurs by means of the mechanisms described above. However, randomized controlled trials (RCTs) of LA investigating cardiovascular mortality have been equivocal and suggest either increased mortality [35] or no overall effect [36]. Such studies are discussed elsewhere [37,38], and it is important to note that they have often employed intakes of LA that are in excess of current recommended intakes that limit total PUFAs to 5 to 10% of energy [39,40]. Furthermore, it seems likely that the interventions with LA were confounded by the presence of significant amounts of trans fats due to the consumption of vegetable oil-based spreads [41]. Despite the apparent failure of RCTs, current evidence supports the potential long-term benefits of LA intake in lowering the risk of CVDs. The latest meta-analysis reports results from 38 studies involving 44 prospective cohorts of 811,069 participants (almost half of the studies were published in the period 2014 to 2020), comparing high versus low dietary LA intakes [42]. The estimated median intake across LA categories ranged from 1.1% to 11.6% of energy among studies that provided adequate data for a dose–response analysis. Higher LA intake, as assessed by dietary surveys or through the use of biomarkers (LA concentration in adipose tissue or blood compartments), was associated with a modestly lower risk of mortality from all causes, CVDs and cancer [42]. The associations between dietary LA intake and mortality from cancer or from all causes were non-linear. Compared with the lowest intake of energy from LA, the relative risk (RR) of total mortality was 0.97 (95% confidence interval (CI) 0.89, 1.06) for 5% of energy intake from LA, and for 10% of energy intake from dietary LA, it was 0.88 (95% CI 0.73, 1.05). For cancer mortality, the RRs were 0.96 (95% CI 0.94, 0.98) and 0.83 (95% CI 0.78, 0.89) for 5% and 10% of energy intake from LA, respectively. When excluding two studies in which participants had cancer at baseline, the RRs of total mortality were 0.94 (95% CI 0.86, 1.02) and 0.81 (95% CI 0.69, 0.96) for 5% and 10% of energy intake from LA, respectively, with the lowest LA intake as the reference. The association between dietary LA intake and CVD mortality was linear; the RR for each 5% increase in energy intake from LA was 0.93 (95% CI 0.91, 0.95).

### 3.4. Linoleic Acid and the Brain

Since it has a low concentration in the brain (<2% of total fatty acids), LA has been considered to be non-functional, especially compared to AA and DHA, which can contribute up to 40% of brain fatty acids [43]. It appears that more than half of LA entering the brain becomes a substrate for β-oxidation or acts as a precursor for the synthesis of oxidized metabolites [44]. The role of these metabolites in the brain is not yet fully understood, although they may be linked to disorders such as migraines. In this context, Ramsden et al. showed that lowering dietary LA from 7% to 2% of energy combined with 1.5 g per day EPA and DHA for three months reduced migraine frequency and improved quality of life [45]. It is not clear whether this is an effect of lower LA, of increased long chain omega-3 PUFAs, or of the combination of the two. Hence, longer-term LA-lowering regimens alone without changing EPA and DHA remain to be investigated for their impact on migraine.

### 3.5. Arachidonic Acid as a Precursor of Bioactive Lipid Mediators

**Figure 4:**
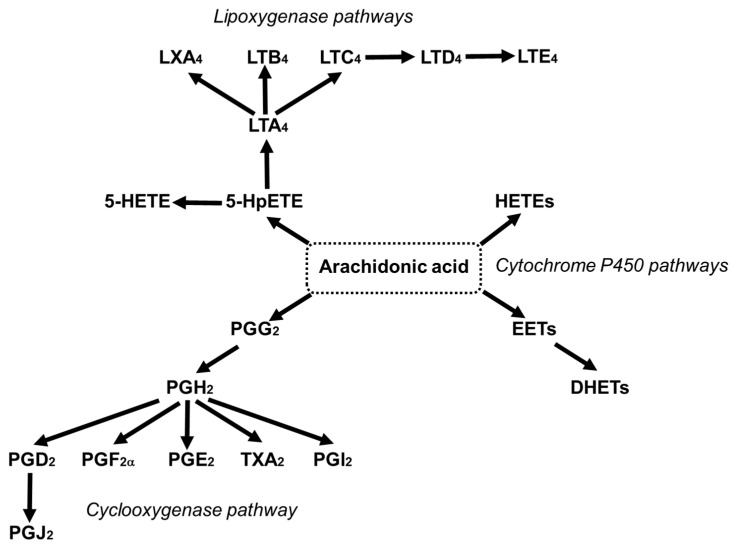
Overview of the cyclooxygenase, lipoxygenase and cytochrome P450 pathways of conversion of arachidonic acid to bioactive mediators. DHET, dihydroxyeicosatrienoic acid; EET, epoxyeicosatrienoc acid; HETE, hydroxyeicosatetraenoic acid; HpETE, hydroperoxyeicosatetraenoic acid; LX, lipoxin; LT, leukotriene; PG, prostaglandin; TX, thromboxane.

AA can contribute up to 25% of the fatty acids in phospholipids of skeletal muscles, brain, liver, platelets, and immune cells [46]. Deacylation and reacylation of AA in cell membranes keeps the level of free AA in cells low and limits its availability for oxidation [47]. The reaction of AA with molecular oxygen through the cyclooxygenase (COX), lipoxygenase (LOX) and cytochrome P450 pathways leads to the generation of mediators which are together referred to as eicosanoids, and include prostaglandins (PGs), thromboxanes (TXs) and leukotrienes (LTs) (Figure 4) [24,48].

Phospholipase A2 can release AA from the membrane into the cell cytosol [49,50]. Physiologically, the majority of AA released is promptly incorporated back into the membrane phospholipids, making the fatty acid unavailable as a substrate for oxidation [51]. Thus, under “resting” conditions, eicosanoid production is low and the metabolites produced have roles in maintaining homeostasis through regulating physiological processes [24]. However, in the presence of some stimuli, for example, inflammatory stimuli, sufficient AA is released to drive significant increases in eicosanoid formation. Hence, in this situation, eicosanoids such as PGD_2_ and E_2_ and 4-series LTs are produced in increased amounts and have roles as mediators and regulators of the inflammatory response [24]. Consequently, many of these eicosanoids are linked to inflammatory disease and multiple anti-inflammatory drugs target the enzymes involved in their synthesis and action [24]. However, it is now recognized that AA-derived metabolites also have roles in the resolution of inflammation: for example, lipoxin A_4_ is a potent pro-resolving mediator [52,53]. Interestingly, the generation of eicosanoids early in the inflammatory response is linked with the later induction of resolution. These considerations have complicated our understanding of the role of omega-6 PUFAs in general, and of AA in particular, in inflammation [54].

In addition to their role in regulating immunity and inflammation [24], AA-derived eicosanoids are involved in regulating platelet aggregation, hemostasis, thrombosis and vascular tone [55,56,57]. Eicosanoids can act as vasodilators (e.g., PGE_2_ and PGI_2_) or vasoconstrictors (e.g., TXA_2_ and the cysteinyl-LTs), therefore affecting blood flow and blood pressure. TXA_2_ and PGI_2_ play a central role in vascular homeostasis and the control of blood flow through their actions on platelets and on vascular smooth muscle cells. TXA_2_ is mainly produced by platelets via COX-1, and its production is increased when platelets are activated. TXA_2_ promotes smooth muscle contraction, acting as a vasoconstrictor, and is a potent activator of platelets, causing their aggregation and leading to thrombus formation. PGI_2_ (sometimes called prostacyclin) is mainly produced by endothelial cells. It is a potent vasodilator and inhibits platelet aggregation and smooth muscle cell proliferation. Hence, the balance in the production of TXA_2_ and PGI_2_ establishes vascular tone and thrombotic potential. PGD_2_ is also an inhibitor of platelet aggregation [58], while the effects of PGE_2_ are related to its concentration [58].

### 3.6. Effects of Increased Intake of Arachidonic Acid

AA is commonly found in foods of animal origin, such as meats, offal (liver, kidney) and eggs. The average daily intake of AA in developed countries is estimated to be in the range of 100 to 350 mg, which is approximately 0.1% of the total energy intake [59]. Additionally, AA can be synthesized from LA (Figure 3). Despite the limited conversion of LA to other omega-6 PUFAs (approximately 1 to 2.2%), it seems that the synthesis of AA from LA is an important metabolic pathway that serves to maintain the level of AA in cell membranes [60,61]. Nevertheless, variations in LA intake over the range seen in Western diets do not result in differences in the AA content of blood or cell lipids [62], and dietary supplementation with 6.5 g/day of LA (as sunflower oil) did not affect the AA content of peripheral blood mononuclear cells (lymphocytes and monocytes) in healthy subjects [63], regardless of LA being a precursor for AA synthesis. The reason for the latter finding may be the already high intake of LA in the Western diet, meaning that the pathway of AA synthesis from LA is already saturated. A systematic review that included the results from 36 RCTs found that neither an increased nor decreased intake of LA relative to the average intake within the Western diet (around 10 g/day) affected AA concentrations in plasma/serum phospholipids or erythrocytes [62].

Supplementation with AA itself increases the content of AA in different blood pools in a dose-dependent manner [59]. This might be expected to affect processes such as inflammation, the immune response and blood clotting, which are all regulated by eicosanoids produced from AA (see earlier). However, a recent systematic review of studies conducted in adults concluded that increasing AA intake to as much as 1000–1500 mg/day had no adverse effect on platelet aggregation and blood clotting, immune function and markers of inflammation [59]. In fact, some benefits of additional AA on cognitive function have been reported [64], which is important especially in the aging population. Furthermore, AA supplementation (600 mg/day) during physical training activities improved performance and increased body strength, peak power, and post-exercise anabolic signaling [65]. It was suggested that 1000 mg/day AA actually decreased inflammation, indicated by lower IL-6, while it increased PGE_2_, which is a potential ergogenic factor, thus increasing tolerance to intense training [66]. Eicosanoids derived from AA promote skeletal muscle growth by controlling differentiation, proliferation and survival of myoblasts, particularly during and after physical activity in healthy subjects [50]. Moreover, AA is a component of some common endocannabinoids that modulate and control neural processes involved in social behavior, pain, and mood [67,68]. Additionally, endocannabinoids are found to regulate gastrointestinal inflammation [69], renal function [70] and sperm motility [71]. Free AA also plays an essential role in neuronal activities acting via ion channels responsible for brain, heart, and muscle cell excitability [72].

### 3.7. Effects of γ-Linolenic Acid and Dihomo-γ-Linolenic Acid

GLA is present in some plant seed oils including evening primrose, blackcurrant, and borage oils (where it contributes about 9%, 17%, and 21% of fatty acids, respectively) [73]. However, its level in typical diets is very low. GLA seems to be efficiently converted to DGLA in many cells and tissues, including some inflammatory cells [73]. GLA is found in low levels in circulating lipids, cells and tissues because of its rapid conversion to DGLA. On the other hand, DGLA is present in circulating lipids and in membrane phospholipids of most cells, and following GLA supplementation, DGLA levels are elevated [63,74,75]. DGLA is a substrate for COX and LOX and its metabolites (e.g., PGE_1_) are reported to mainly have anti-inflammatory properties and to inhibit platelet aggregation [73]. Hence, the ratio of AA/DGLA in the circulation, cells or tissues may influence inflammatory processes and thrombosis. The differential enzymatic activities of cells and tissues to elongate GLA to DGLA and then desaturate DGLA to AA will determine the ratio of AA/DGLA and thus the balance of their metabolites. Some inflammatory cells, such as human neutrophils, seem to have high elongase relative to desaturase activity, but many other tissues (brain, intestine, liver, kidney) appear to have both activities [73]. Interestingly, supplementation with GLA leads to elevated DGLA but not AA in specific inflammatory cells, whereas both DGLA and AA levels are increased in circulating lipids and some tissues [73].

## 4. Omega-3 PUFAs and Human Health

### 4.1. DHA and Development of the Brain and Eye

Both EPA and DHA have important functions in the body [76]. DHA has many roles: it participates in regulating the active transport of amino acids (choline, glycine, taurine) through the cell membrane [77,78], in regulating the function of sodium channels [72], and in initiating the response of rhodopsin to visual stimuli [79]. DHA is the most abundant omega-3 fatty acid in the central nervous system and retina [80], which is not surprising given its roles: it is important for neurotransmission, neuroplasticity and signal transduction [81,82,83,84]. DHA increases serotonin and acetylcholine concentration in nervous tissue [85] and is a precursor of unique modulatory molecules, including (neuro) protectin D1 [86]. DHA appears to be essential, not only for the functioning and maintenance of the visual and nervous systems, but also for prenatal and early postnatal brain and visual development [80,87]. In this context, there is evidence to suggest that omega-3 PUFA supplementation during pregnancy is linked to cognitive and visual development and brain function in the offspring [80,85]. However, a systematic review of 11 RCTs of maternal omega-3 PUFA supplementation during pregnancy suggested few differences in cognitive, language, or motor development between the groups, except for cognitive scores in 2- to 5-year-old children, in whom maternal omega-3 supplementation resulted in higher developmental standard scores [88]. Nevertheless, because of the strength of the evidence for the early roles of DHA in cognitive and visual development, the European Food Safety Authority has mandated the inclusion of DHA in infant formula [89].

### 4.2. EPA and DHA and Psychological, Psychiatric and Behavioral Disorders

Although historically there was much focus on the role of DHA in early cognitive and visual development, due its high content in the brain and visual system, omega-3 PUFAs are likely to have important roles in the brain beyond infancy and are probably important for brain function throughout the life course [85,90,91,92,93].

A number of studies have reported lower levels of EPA and DHA in the bloodstream of children with attention deficit hyperactivity disorder or autistic spectrum disorders than in control children [94] and it has been postulated that normalizing the fatty acid levels would result in clinical benefit in these conditions. This has been examined in quite a number of trials in children and adolescents with attention, learning, or behavioral disorders, some showing some improvements but others finding no effect (see [76] for References). These trials have been reviewed and subjected to meta-analysis many times, with differing conclusions [95,96,97,98,99]. The different findings most likely relate to several factors that differ between the studies, including the dose of omega-3 PUFAs used, the balance of EPA to DHA, the duration of supplementation, the precise outcome(s) measured, and differences in the children studied. One review concluded that studies using higher doses of omega-3 PUFAs or of longer duration or in children/adolescents with low socioeconomic status were more likely to demonstrate benefits [95].

Data from nine countries were used to demonstrate a significant inverse correlation between annual fish consumption and the prevalence of major depression [100]. This observation is compatible with a proposed protective effect of omega-3 PUFA towards depression. Many observational studies have investigated the relationship between fish and omega-3 fatty acid intake and depression; such studies have been subject to meta-analysis [101]: compared with the lowest intake of EPA + DHA, the highest intake was associated with a relative risk of depression of 0.82 (95% CI 0.73, 0.92). There have also been a number of RCTs of EPA + DHA in individuals with depression. For example, a small study using a very high dose of EPA + DHA (9·6 g/day) reported a decrease in depressive symptoms [102], while this effect was not seen in a study using a lower dose of DHA alone (2 g/day) [103]. EPA + DHA (6.2 g/day) given for 4 months significantly improved almost all outcomes, including depressive symptoms, in patients with bipolar manic depression [104]. Similarly, EPA (2 g/day for 4 weeks) improved symptoms in patients with unipolar depressive disorder [105]. There are many meta-analyses of RCTs involving participants with depressive disorders, including a number published fairly recently [106,107,108,109,110,111]. One analysis of 26 studies, which included 2160 participants, identified an overall benefit from EPA + DHA [108]. Furthermore, the analysis suggests that use of pure EPA formulations or formulations containing >60% EPA was associated with clinical benefits, whereas pure DHA or DHA-rich formulations did not show benefits. Another meta-analysis of 20 RCTs also identified significant benefit from omega-3 PUFAs [109] but concluded that better quality evidence is needed. Another recent meta-analysis identified no significant effect of EPA + DHA on depressive symptoms in older people (>65 years of age) who “were well”, while there was “a large effect” of EPA + DHA in older people with depression [110]. It was concluded that “omega-3 PUFAs are effective in the treatment of elderly depressed patients” [110]. Despite these findings, it is important to note that some recent meta-analyses of RCTs of EPA + DHA in depression do not find evidence of benefit [111]. Nevertheless, the International Society for Nutritional Psychiatry Research supports the use of omega-3 PUFAs for the prevention of depression in high-risk populations and for the treatment of depression in pregnant women, children, and the elderly [112]. It is suggested that either pure EPA or a combination of EPA + DHA with a ratio of at least 2 in favor of EPA be used, while the suggested EPA dose is 1 to 2 g/day.

Patients with schizophrenia have lower levels of EPA and DHA in their erythrocytes than do controls (see [76] for References). The first trial of omega-3 PUFAs in schizophrenia identified clinical improvement with EPA (2 g/day), but not with DHA [113], while subsequent trials also showed benefit with EPA [114,115], but not all studies have seen this [116]. Another study reported significant benefits of omega-3 PUFAs given for 26 weeks to patients with schizophrenia [117]. Despite these findings, a Cochrane review concluded that omega-3 PUFAs should be regarded only as an experimental treatment for schizophrenia [118]. A study reported significant benefit from 1 g/day EPA in borderline personality disorder [119], while a small number of studies report anti-aggressive effects of DHA [120,121].

### 4.3. EPA and DHA and Cognitive Decline

A meta-analysis of observational studies showed a positive association between dietary intake or plasma levels of DHA and memory in adults [122]. An inverse association between DHA intake and risk of dementia and Alzheimer’s disease was identified through a meta-analysis of observational studies [123]. Post-mortem studies have reported that the brains of people with Alzheimer’s disease contain less DHA than those without the disease [124,125,126], and some studies have also linked low levels of omega-3 PUFAs in the blood to dementia [127,128,129]. Higher plasma phosphatidylcholine DHA was associated with a 47% lower risk of developing all-cause dementia and a 39% lower risk of Alzheimer’s disease in a subgroup of the Framingham Heart Study [130].

Sinn et al. [131] reported that EPA + DHA (1.8 g/day for 6 months) reduced depressive symptoms and improved cognition in adults with mild cognitive impairment. However, compared with placebo, daily supplementation with 1.7 g DHA and 0.6 g EPA for 6 months did not affect the Mini-Mental State Examination score in patients with Alzheimer’s disease being treated with an acetylcholine esterase inhibitor [132]. Nevertheless, the omega-3 PUFAs had a significant effect on cognitive functioning measured with the Alzheimer’s Disease Assessment scores as well as the sub-items, and a correlation was found with the increase in plasma omega-3 PUFAs [133]. This suggests that the effect of omega-3 PUFAs depends on the specific aspect of cognitive health assessed. Furthermore, subgroup analysis showed a benefit of omega-3 PUFAs in the group with very mild cognitive decline at baseline [132]. A meta-analysis of six RCTs of duration 3–40 months and using 0.14 to 1.8 g EPA + DHA daily identified a slower rate of cognitive decline in those receiving omega-3 fatty acids [134], while a systematic review reached the conclusion that the most beneficial effect of EPA and DHA supplementation in Alzheimer’s patients can be expected in the early stage of the disease [135]. These findings all suggest that individuals with mild cognitive decline would be a good target group for omega-3 PUFAs. However, it might be even more beneficial to start intervention with omega-3 PUFAs before any cognitive decline occurs [136]. In this context, it has been shown that omega-3 PUFA supplementation in healthy older people has a beneficial effect on white matter microstructural integrity, grey matter volume in specific brain areas and vascular parameters accompanied by improved executive function [137]. This indicates that there might be a potential for preventive uses of omega-3 PUFAs to maintain cognitive health in older people.

### 4.4. EPA and DHA and Inflammation

Both EPA and DHA have a range of anti-inflammatory effects [11,138,139,140]. Primarily, EPA and DHA decrease the production of AA-derived eicosanoids. They do this partly by competing with AA for incorporation into cell membrane phospholipids (i.e., EPA and DHA result in lower amounts of AA in cell membranes), partly by reducing the release of AA from membranes, partly by inhibiting the action of the enzymes COX-2 and 5-LOX on AA, and partly by competing with AA for metabolism by COX and LOX enzymes [138]. Intervention studies have shown that increased intake of EPA + DHA results in increased concentrations of EPA and DHA in the membranes of cells involved in inflammation (and most likely in many other cell types) (see [138] for References]. As well as being linked with decreased production of AA-derived eicosanoids, this increase in omega-3 PUFA content is linked with decreased levels of other inflammatory markers including various cytokines and chemokines, acute-phase proteins, and adhesion molecules [11,138,139,140,141]. For example, a number of human studies show inverse associations between EPA and DHA status (e.g., omega-3 index which is the sum of EPA plus DHA in erythrocytes) and blood markers of inflammation such as C-reactive protein [142,143,144], cytokines such as IL-6 [142,143,144] and adhesion molecules such as soluble intercellular adhesion molecule 1 [143]. Furthermore, meta-analyses of RCTs with EPA and DHA confirm reductions in the concentrations of C-reactive protein [145,146] and several cytokines [147].

The anti-inflammatory effects of EPA and DHA are often reported to involve decreased activation of the pro-inflammatory transcription factor nuclear factor kappa-light-chain-enhancer of activated B cells (NFκB) in response to inflammatory stimuli as a result of the inhibition of phosphorylation of the inhibitory subunit of NFκB, IκB [148,149]. Some studies have linked this effect to membrane-mediated actions of EPA and DHA that inhibit the early stages of inflammatory signaling [150]. These actions require EPA and DHA to be incorporated into cell membrane phospholipids. However, it appears that EPA and DHA can also act directly on inflammatory cells via membrane receptors to diminish inflammatory responses. GPR120 is a plasma membrane G protein coupled receptor (also called free fatty acid receptor 4) that is able to bind long-chain fatty acids, especially DHA [151]. GPR120 shows high expression on adipocytes and inflammatory cells such as macrophages [151]. DHA activation of GPR120 was shown to reduce NFκB activation in macrophages and to decrease the production of inflammatory cytokines [151]. Recent updates on omega-3 PUFA actions mediated through GPR120 can be found elsewhere [152,153,154,155]. This mechanism of action means that EPA and DHA can exert anti-inflammatory effects that do not require their incorporation into cell membranes and do not involve modification of lipid mediator production.

**Figure 5:**
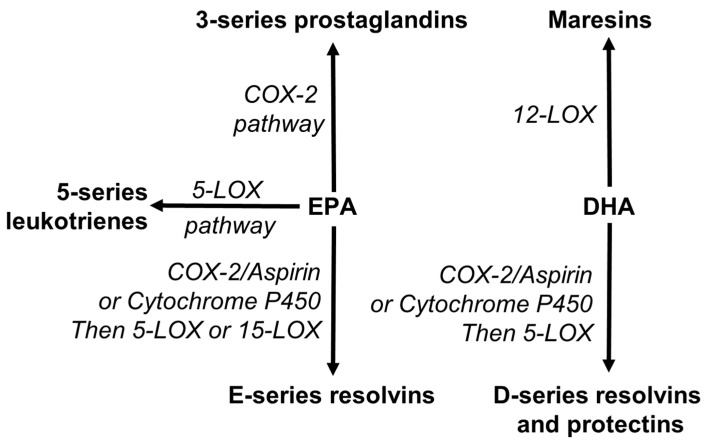
Overview of the conversion of eicosapentaenoic acid (EPA) and docosahexaenoic acid (DHA) to lipid mediators, including specialized pro-resolving mediators (resolvins, protectins, maresins). COX, cyclooxygenase; LOX, lipoxygenase.

EPA and DHA are also recognized as precursors for the synthesis of novel specialized pro-resolving mediators (SPMs). SPMs include resolvins, protectins and maresins [53,156,157,158]. Resolvins are synthesized from both EPA (E-series) and DHA (D-series), while maresins and protectins are synthesized from DHA (Figure 5). These pathways use the enzymes involved in the pathways of synthesis of eicosanoids (Figure 5).

As their name indicates, SPMs activate the resolution of inflammation. Hence, a lack of the omega-3 PUFAs, especially EPA and DHA, may not favor resolution of inflammation and may, in fact, promote the pathogenesis of various diseases in which inflammation is involved. Increased intake of EPA and DHA has been reported to result in higher concentrations of some SPMs in human blood and tissues (see [159] for References).

As a result of their various interacting anti-inflammatory and inflammation-resolving actions, increased intake of EPA and DHA could have therapeutic potential in diseases involving inflammation. This has been well explored in RCTs in rheumatoid arthritis where high doses of these omega-3 PUFAs induce several clinical benefits [160,161,162]. Systematic reviews of the use of EPA and DHA in patients with rheumatoid arthritis confirm a reduction in pain [163,164]. An observational study reported that a higher blood level of EPA was associated with greater treatment efficacy of anti-tumor necrosis factor antibodies in patients with rheumatoid arthritis [165]. The anti-inflammatory effects of EPA and DHA are likely to extend beyond “classic” chronic inflammatory diseases into age-related conditions characterized by low-grade inflammation such as CVDs, metabolic disease, cognitive decline, and sarcopenia [140,166,167]. However, the possible benefits of omega-3 PUFAs on these latter conditions will also involve effects on several processes in addition to inflammation. For example, EPA and DHA beneficially modify blood lipids [168,169], and platelet aggregation [170,171], contributing to lower risk of CVDs. The effects on blood lipids relate to the regulation of pathways of lipid synthesis and degradation partly via the action of transcription factors and gene expression, especially in the liver [172], while the effects on platelet aggregation relate to modification of eicosanoid profiles [24], as reviewed elsewhere recently [173]. In addition, omega-3 PUFAs play an important role as antioxidants and regulate antioxidant signaling pathways. Mitochondrial membranes have a high DHA content, and research suggests that DHA is crucial for adenosine triphosphate synthesis by oxidative phosphorylation [10]. DHA is reported to reduce mitochondrial oxidative stress and cytochrome c oxidase activity while increasing manganese-dependent superoxide dismutase activity [10]. The anti-inflammatory effects of omega-3 PUFAs may also be important in this regard, since inflammation induces oxidative stress (Figure 1).

### 4.5. EPA and DHA and CVDs

The long history of omega-3 PUFAs, especially EPA and DHA, and CVDs has been reviewed in detail elsewhere [174,175,176,177,178]. Results from both cohort studies and RCTs have been subject to systematic review and meta-analysis numerous times (see [178] for a summary). For example, Chowdhury et al. [179] brought together prospective studies that examined the association of dietary or circulating fatty acids with risk of coronary outcomes. Aggregation of data from 16 cohort studies involving over 422,000 individuals showed a 13% lower risk for those in the top third of dietary intake of EPA + DHA compared with those in the lower third of intake [179]. Data from 13 studies involving over 20,000 individuals showed 22%, 21% and 25% lower risk of coronary outcomes for those in the top third of blood levels of EPA, DHA and EPA + DHA, respectively, compared with those in the lower third [179]. Alexander et al. [180] reported data from prospective cohort studies reporting the association of dietary EPA and DHA with risk of various coronary outcomes. The aggregation of data from 17 studies showed an 18% reduction in risk for any coronary heart disease event for those with higher dietary intake of EPA + DHA compared to those with lower intake. There were also significant reductions of 23%, 19% and 47% in the risk for fatal coronary events, coronary death and sudden cardiac death, respectively. Another study aggregated data from 19 studies that investigated the association between EPA or DHA concentration in a body pool such as plasma, serum, erythrocytes or adipose tissue and risk of future CHD in adults who were healthy at study entry [181]. EPA and DHA were each associated with a lower risk of fatal CHD, with about a 10% lower risk for each one standard deviation increase in either EPA or DHA. Harris et al. [182] used data from 10 cohort studies and identified a 15% reduction in risk of fatal CHD for each one standard deviation increase in the omega-3 index. Clearly, there is substantial evidence that omega-3 PUFAs, especially EPA and DHA, reduce the risk of CVDs, especially CHD [178], acting through plausible mechanisms of action to beneficially affect a range of risk factors [145]. Thus, omega-3 PUFAs, especially EPA and DHA, have a role in the prevention of CVDs; this is the basis of most recommendations for the intake of EPA and DHA.

Despite the evidence in favor of a preventative role of EPA and DHA towards CVDs, the effect of EPA and DHA as a treatment in patients at increased risk of CVDs (e.g., post myocardial infarction) remains controversial, partly because of inconsistent findings in large trials [178,183]. These inconsistent findings have meant that outcomes from meta-analyses of this literature base have changed several times over the last 20 years (see [178] for a summary). Nevertheless, findings of a 2019 meta-analysis of 13 RCTs [184] and of a 2021 meta-analysis of 40 trials [185] indicate a dose-dependent therapeutic effect of EPA and DHA: a statistically significant inverse linear dose–response relationship was found between EPA + DHA administration and risk of CVD outcomes [184,185]. It was estimated that every 1 g/day EPA + DHA corresponded to a 9% and 7% lower risk of myocardial infarction and total coronary heart disease, respectively, and to a 5.8% lower risk of CVD events. The Reduction of Cardiovascular Events with Icosapent Ethyl Intervention Trial (REDUCE-IT) [186] involved patients with established cardiovascular risk or with diabetes and elevated triglyceride level (1.52 to 5.63 mmol/L), who were receiving statin therapy. In the trial, they received 4 g/day of a formulation rich in EPA ethyl ester (providing 3.6 g of EPA daily). Mineral oil was used as a placebo. The median duration of follow-up was 4.9 years. The primary outcome was a composite of cardiovascular death, nonfatal myocardial infarction, nonfatal stroke, coronary revascularization or unstable angina. The key secondary end point was a composite of cardiovascular death, nonfatal myocardial infarction, or nonfatal stroke. Compared with placebo, EPA resulted in a statistically significant reduction in both the primary and secondary outcomes, as well as a number of other clinical outcomes [186]. Another recent trial, the Effect of Vascepa on Improving Coronary Atherosclerosis in People with High Triglycerides Taking Statin Therapy (EVAPORATE) trial [187] used the same EPA ethyl ester preparation and the same dose as used in REDUCE-IT. EVAPORATE demonstrated that EPA might directly promote atherosclerotic plaque regression in hypertriglyceridemic individuals [187]. Thus, omega-3 PUFAs may directly target atherosclerotic plaques in patients who already have advanced CVD, as suggested by earlier studies in mice [188] and humans [189,190]. The positive findings of REDUCE-IT and EVAPORATE contrast with those of the more recently published Long Term Outcomes Study to Assess Statin Residual Risk with Epanova in High Cardiovascular Risk Patients with Hypertriglyceridemia (STRENGTH) trial [191]. In this trial, statin-treated patients with hypertriglyceridemia and high cardiovascular risk were given 4 g/day of a formulation of highly purified EPA and DHA as free fatty acids (providing approximately 2.2 g EPA and 0.8 g DHA daily) or corn oil as placebo. There was no significant difference in a composite outcome of major adverse cardiovascular events between the groups and the trial was stopped early [191]. Reasons for the differences in the findings of REDUCE-IT and STRENGTH are discussed elsewhere [183] and will be summarized here. One explanation could be that EPA and DHA have differential effects on some cardiometabolic risk factors [192], and therefore a formulation of pure EPA could have a different effect from a formulation that combines EPA and DHA. Both EPA and DHA lower triglycerides [192], an effect seen in both REDUCE-IT and STRENGTH to a similar extent (decrease of 18%). DHA is reported to increase LDL-cholesterol more than EPA does [192]. This could explain why LDL-cholesterol was increased by 3% with EPA + DHA treatment in STRENGTH while LDL-cholesterol was lowered by an average of 6.6% in REDUCE-IT which provided EPA but not DHA. DHA increases HDL-cholesterol, especially the HDL2 subfraction which is cardioprotective, but EPA does not affect HDL-cholesterol [192]. However, both REDUCE-IT and STRENGTH reported higher HDL-cholesterol with omega-3 PUFAs. One final difference between REDUCE-IT and STRENGTH is the choice of placebo, mineral oil in REDUCE-IT and corn oil in STRENGTH. It has been argued that mineral oil may have adverse effects on some cardiovascular risk factors [193], therefore making omega-3 PUFAs appear beneficial, as in REDUCE-IT. A recent systematic review of mineral oil when used as a placebo indicated inconsistent effects on blood lipids and other biomarkers and reached the conclusion that mineral oil is not likely to be responsible for effects seen in trials like REDUCE-IT and EVAPORATE [194]. Thus, at this point in time, the exact reasons for the different findings of REDUCE-IT and STRENGTH are not clear.

### 4.6. α-Linolenic Acid and Human Health

The health benefits of EPA and DHA may be achieved by increasing intake of oily fish or by using supplements that contain EPA and DHA. However, the question of whether the major plant omega-3 PUFA, ALA, possesses these same benefits, either in its own right, or following conversion to EPA and DHA is an important one [14]. ALA has been demonstrated to have anti-inflammatory, neuroprotective, and antidepressant effects [14,195]. Increasing ALA intake may produce modest cardioprotection by lowering total and LDL-cholesterol (relative to saturated fatty acids), maintaining endothelial function, and through antithrombotic and anti-inflammatory effects [196,197]. Human studies on the effects of ALA often use high intakes and report some conversion to EPA, although not to DHA [14]. In fact, the conversion efficiency of ALA is low in human adults; it estimated that only 5% of ALA is converted to EPA and less than 1% to DHA [198]. Thus, any protective effects of ALA are likely due to ALA itself, unless high intakes are used in which case they may be due to EPA [14].

## 5. Relevance of PUFAs to COVID-19

Since late 2019, a novel infection with severe acute respiratory syndrome coronavirus 2 has resulted in a global pandemic [199]. Symptoms of the resulting coronavirus disease discovered in 2019 (COVID-19) are highly variable, and range from none to a life-threatening illness that is related to the strength of the immune-inflammatory response of infected individuals [200]. Uncontrolled release of pro-inflammatory cytokines and excessive coagulation usually accompany this acute respiratory disease [200,201]. Therefore, current treatments are focused on addressing inflammation and thrombosis. As mentioned already, omega-3 fatty acids, especially EPA and DHA, are anti-inflammatory, promote synthesis of pre-resolving mediators and regulate platelet aggregation and thrombosis. These effects suggest that EPA and DHA might be useful as part of therapy for COVID-19 [202]. Omega-3 fatty acids are found to upregulate some of the functions of cells that are part of the innate immune response, including neutrophils, macrophages, natural killer cells, mast cells, basophils, and eosinophils. They also promote antigen-specific responses mediated by T-cells and B-cells, producing antibodies and generating an immunological memory specific to repeated infection with the same pathogen [203]. Higher COVID-19-related deaths have been seen in the regions with a low omega-3 index, such as the USA and some European countries [202]. However, this is simply an association with no demonstration of a cause and effect relationship. A pilot study published by Asher et al. reported on how the omega-3 index related to COVID-19 outcomes in 100 patients [204]. The critical endpoint was death from COVID-19 infection. In models adjusted for age and sex, it was found that patients with an omega-3 index higher than 5.7% had about 75% lower risk for mortality relative to those below that value. A randomized clinical trial published the effects of omega-3 fatty acid supplementation on multiple outcomes in critically ill patients with COVID-19 [205]. A fourteen-day intervention with EPA and DHA (400 and 200 mg daily added to the enteral feed) resulted in significantly better one-month survival compared with the control group (21% vs. 3%). EPA + DHA also improved markers of kidney function and some markers of respiratory function and increased blood lymphocyte numbers; however, it is important to note that many other outcomes were not different between groups. Nevertheless, this trial gives encouragement for EPA + DHA as a therapeutic option [206]. In this regard, it is noteworthy that the dose of EPA and DHA used by Doaei et al. [205] is modest compared with doses used in many trials in patients with CVDs or inflammatory conditions. Thus, stronger effects might be seen at high doses. Whether omega-3 PUFAs can reduce the incidence of coronavirus infection itself is not clear. However, results from a COVID-19 Symptom Study app using results from 445,850 users indicate a modest reduction in the risk of testing positive for infection in those who use omega-3 PUFA supplements [207].

## 6. Conclusions

PUFAs display a plethora of biological activities at the molecular and cellular levels that result in effects on cell and tissue function linked to health outcomes and disease risk. Antioxidant and anti-inflammatory properties of omega-3 PUFAs are important and are recognized as contributing to reduced disease risk and severity (i.e., better health). Furthermore, these properties offer therapeutic opportunities in conditions marked by excessive inflammation and oxidative stress. Both omega-6 and omega-3 PUFAs lower CVD risk through multiple interacting mechanisms; the omega-6 PUFA LA acts mainly through lowering LDL-cholesterol, while both the omega-3 PUFAs EPA and DHA lower triglycerides, promote blood flow and cardiac and vascular function and control thrombosis and inflammation. The actions of pro-resolution mediators produced from EPA and DHA are particularly important. The breadth of the effects of omega-3 PUFAs explains why they have roles in both the prevention and treatment of CVDs, especially of CHD, and in the control of inflammatory conditions and various neurological disorders. Although the eicosanoid mediators produced from the omega-6 PUFA AA are mainly pro-inflammatory in character, current evidence shows only limited effect of LA and AA on inflammatory biomarkers at intakes currently consumed in human diets. Furthermore, AA, similar to DHA, is important in brain development and cognitive function. The relationship between omega-6 and omega-3 PUFAs and their bioactive lipid mediators in the context of inflammation is not yet fully understood. It is widely accepted that an optimal ratio of omega-6 and omega-3 PUFAs within a healthy diet positively affects inflammation and other biological processes, although there is no consensus on what that ratio should be. Likewise, the precise balance between the omega-3 PUFAs EPA and DHA for optimal health is unclear, although both seem to be important. DHA is vital for early life visual and neural development. The principal roles of the plant omega-3 PUFA ALA are in controlling conversion of LA to AA and in acting as a substrate for synthesis of EPA. Based on current early evidence, omega-3 PUFAs (EPA + DHA) have promising effects in COVID-19 patients, although further clinical studies are needed to confirm these benefits.

## Data Availability

Not applicable.
